# Human brain tissue identification using coherent anti-Stokes Raman scattering spectroscopy and diffuse reflectance spectroscopy for deep brain stimulation surgery

**DOI:** 10.1117/1.NPh.11.2.025006

**Published:** 2024-06-12

**Authors:** Sébastien Jerczynski, Mireille Quémener, Valérie Pineau Noël, Antoine Rousseau, Elahe Parham, Alexandre Bédard, Shadi Masoumi, Thomas Charland, Anthony Drouin, Jonathan Roussel, Valérie Dionne, Thomas Shooner, Anaïs Parrot, Mohamad A. Takech, Éric Philippe, Damon DePaoli, Léo Cantin, Martin Parent, Daniel C. Côté

**Affiliations:** a CERVO Brain Research Center, Québec City, Québec, Canada; b Centre d’optique, photonique et laser, Québec City, Québec, Canada; c Centre Hospitalier de l’Université Laval, CHU de Québec-Université Laval, Québec, Canada; d Laboratoire d’anatomie, Faculté de médecine de l’Université Laval, Québec, Canada; e Hôpital de l’Enfant-Jésus, CHU de Québec-Université Laval, Québec, Canada

**Keywords:** Parkinson’s disease, deep brain stimulation, coherent anti-Stokes Raman scattering, diffuse reflectance spectroscopy, optical guidance

## Abstract

**Significance:**

We assess the feasibility of using diffuse reflectance spectroscopy (DRS) and coherent anti-Stokes Raman scattering spectroscopy (CARS) as optical tools for human brain tissue identification during deep brain stimulation (DBS) lead insertion, thereby providing a promising avenue for additional real-time neurosurgical guidance.

**Aim:**

We developed a system that can acquire CARS and DRS spectra during the DBS surgery procedure to identify the tissue composition along the lead trajectory.

**Approach:**

DRS and CARS spectra were acquired using a custom-built optical probe integrated in a commercial DBS lead. The lead was inserted to target three specific regions in each of the brain hemispheres of a human cadaver. Spectra were acquired during the lead insertion at constant position increments. Spectra were analyzed to classify each spectrum as being from white matter (WM) or gray matter (GM). The results were compared with tissue classification performed on histological brain sections.

**Results:**

DRS and CARS spectra obtained using the optical probe can identify WM and GM during DBS lead insertion. The tissue composition along the trajectory toward a specific target is unique and can be differentiated by the optical probe. Moreover, the results obtained with principal component analysis suggest that DRS might be able to detect the presence of blood due to the strong optical absorption of hemoglobin.

**Conclusions:**

It is possible to use optical measurements from the DBS lead during surgery to identify WM and GM and possibly the presence of blood in human brain tissue. The proposed optical tool could inform the surgeon during the lead placement if the lead has reached the target as planned. Our tool could eventually replace microelectrode recordings, which would streamline the process and reduce surgery time. Further developments are required to fully integrate these tools into standard clinical procedures.

## 1 Introduction

Parkinson’s disease (PD) is the second most common neurodegenerative disorder after Alzheimer’s disease.^1^ The primary clinical signs of PD are motor symptoms that include resting tremors, muscular rigidity, and slowness of movements.^2^ The initial treatment of PD usually starts by administering the dopamine precursor l-dopa. However, over time, this pharmacological treatment becomes less effective, and most patients will eventually suffer from l-dopa-induced dyskinesia.^3^^,^^4^ For these patients, deep brain stimulation (DBS) surgery is often the recommended treatment option.^5^

DBS is widely recognized as the most effective surgical treatment for movement disorders, in particular PD.^6^^,^^7^ The surgery targets a specific brain region with an electrode that emits high-frequency electrical pulses,^8^ leading to a significant reduction in PD motor symptoms and improving quality of life. The target regions are deep subcortical nuclei that are components of the basal ganglia. According to the progression of the disease and hospital protocols, the optimal target region for PD is either the subthalamic nucleus (STN) or the internal globus pallidus (GPi).^9^ Since those targets are only a few millimeters in size, a high level of expertise as well as precise navigation tools are needed for accurate DBS lead placement.

Current DBS procedures require three key steps before the surgery can begin: (1) localize the target region in the patient’s brain, (2) determine the entry point, and (3) plan the trajectory to the target. Those steps are commonly performed preoperatively using magnetic resonance imaging (MRI) registered with stereotactic atlases.^10^ After planning the trajectory to reach the target, the surgery can take place. The first step is to drill a burr hole to open the skull. However, drilling a burr hole can lead to cerebrospinal fluid leakage, possibly resulting in brain shifts greater than 2 mm,^11^^–^^13^ which is significant considering the small size of the DBS targets. The consequences of lead misplacements as little as 1 mm for patients are important: it might cause undesirable side effects such as paresthesia, oculomotor disturbances, affective changes, dysarthria, and neuropsychological disorders such as depression that might require additional surgery.^14^^–^^16^ In 2000, a study demonstrated that ∼45% of DBS surgeries resulted in DBS lead misplacements of over 3 mm from the originally intended target.^17^ Another study showed that 41% of PD patients who complained about suboptimal surgery outcomes had a misplaced DBS lead and that repositioning the lead by only 1 mm improved patient outcomes significantly.^18^

In some health centers, to help minimize the impact of brain shifts, microelectrode recordings (MERs) are used during the surgery to map the brain along the planned trajectory and confirm the target location prior to lead insertion.^19^^–^^21^ However, this microelectrode is pulled out before the stimulation lead is inserted in the MER-identified target, and there are no tools to assess the placement of the stimulation lead as it is being placed. In addition, the use of intraoperative MERs itself does not always provide better surgical outcomes.^14^^,^^22^ For example, the presence of active neurons in white matter (WM) and electrically silent neurons in gray matter (GM) bias MER interpretations.^22^^,^^23^ Finally, MER is a time-consuming technique^24^ that is not available at all health centers. Providing neurosurgical guidance of the stimulation lead from real-time *in situ* measurements could streamline the surgical process for both the surgical team and the patient, decrease the surgery time, reduce the overall treatment cost, and prevent adverse neuropsychological consequences.

Optical techniques are well suited to address the need for real-time feedback and have already shown potential for implementation in clinics.^25^^,^^26^ Past studies from our research laboratory have introduced two fiber-based methods of choice for neurosurgical guidance: wavelength-swept coherent anti-Stokes Raman scattering (CARS) spectroscopy ^27^ and diffuse reflectance spectroscopy (DRS).^28^ CARS is a label-free spectroscopy technique capable of probing unique molecular bonds. CARS is suitable for brain sensing and imaging due to the abundance of ${\mathrm{CH}}_{2}$ bonds in myelinated axons. This abundance provides a strong and molecularly specific contrast between WM and GM. Using a wavelength-swept source for CARS is more appropriate for highly scattering, thick tissues compared with a spectrometer-based measurement.^29^ DRS takes advantage of low-cost broadband light sources to quantify the scattering and absorption properties of various brain structures (more details in Ref. 29).

Here, we show the first use of these spectroscopic methods for brain tissue identification in a fresh human cadaver during DBS surgery. We performed the measurements inside a DBS lead by replacing a commercial rigid stylet with another custom stylet housing fiber optics. The enhanced lead was inserted to target the STN, the GPi, and one off-target region, in both hemispheres. The optical measurements were taken within a functional operating room, using our custom, mobile optical console. The tissue was identified using a spectral analysis dimensionality reduction technique called principal component analysis (PCA) with the k-means clustering method to classify each spectrum as either WM or GM for each point along the trajectory. To assess the viability of our collection of optical measurements, we analyzed histological slices. Our findings suggest that optical measurements have the potential to provide enough information for real-time brain tissue identification, opening the door to neuronavigation in human DBS surgeries. Altogether, this work provides a clinically relevant proof-of-concept approach with no additional invasiveness.

## 2 Materials and Methods

Postmortem material was gathered from an 85-year-old man who died from metastatic prostate neoplasia, without any evidence of neurological, neurodegenerative, or psychiatric diseases. The postmortem material was obtained from the Human Anatomy Laboratory at Université Laval, and the brain was stored in the Human Brain Bank at CERVO Research Center. Informed consent was obtained from the donor before tissue donation by both institutions. Local ethics committees (Institut universitaire en santé mentale de Québec, 2013-3, 146-2012) approved collection procedures, storage, and handling of postmortem material. All methods were executed following relevant guidelines and regulations.

At 24 postmortem hours, the head was placed in phosphate-buffered saline (PBS). The surgery was performed 8 days later by a neurosurgeon at Hôpital de l’Enfant-Jésus (Québec, Canada), who followed the standard DBS protocol normally reserved for patients with PD. The brain tissue was not chemically preserved prior to the experiment.

### 2.1 Trajectory Planning and Preoperative MRI

Preoperative three Tesla MRI scans (Siemens MAGNETOM Skyra, Munich, Germany) of the head were acquired [T1-weighted, T2-weighted, and fluid-attenuated inversion recovery (FLAIR) sequences] with a stereotactic frame (CRW, Radionics) that was used by the neurosurgeon to plan the lead insertion for six targets (STN, GPi, and another off-target region in both hemispheres). T1-weighted sequence main parameters are the following: 2000 ms repetition time, 8.5 ms echo time, 860 ms inversion time, and 150 deg flip angle.

**Fig. 1:**
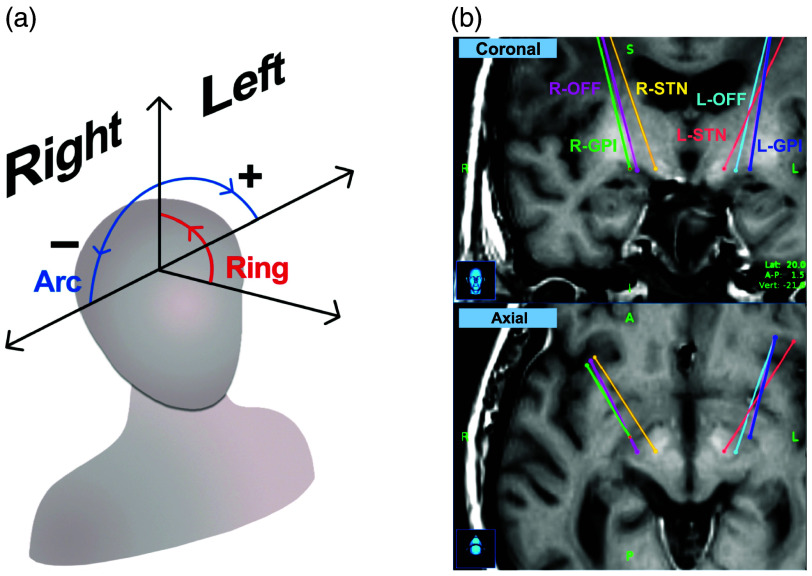
Trajectory planning prior to the DBS surgery. (a) Coordinate system used for the insertion planning. The arc angle is positive for the left hemisphere and negative for the right hemisphere. (b) Insertion angles determined with the Medtronic StealthStation™ neuronavigation system using the preoperative MRI scans for the six trajectories.

The StealthStation neuronavigation software was used to plan the six trajectories using the built-in brain atlas, to calculate the insertion angles, and to determine the target coordinates. These parameters were then used for the lead insertions. The two angles (ring and arc) are shown in relation to the position of the head in Fig. 1(a), and the values are listed in Table 1. The insertion trajectories can be visualized on the preoperative MRI scan in Fig. 1(b). Note that, throughout the paper, we refer to the six trajectories by the hemisphere side (R: right, L: left) followed by the targeted region (STN, GPi, and OFF for the off-target region).

**Table 1:** Insertion angles determined with the Medtronic StealthStation™ neuronavigation system for the six trajectories.

Target	R-STN	R-GPi	R-OFF	L-STN	L-GPi	L-OFF
Ring angle (deg)	57.6	63.3	60.1	50.7	54.5	51.2
Arc angle (deg)	−16.8	−12.7	−12.1	20.2	7.4	11.1

### 2.2 Surgery and Spectroscopic Measurements

**Fig. 2:**
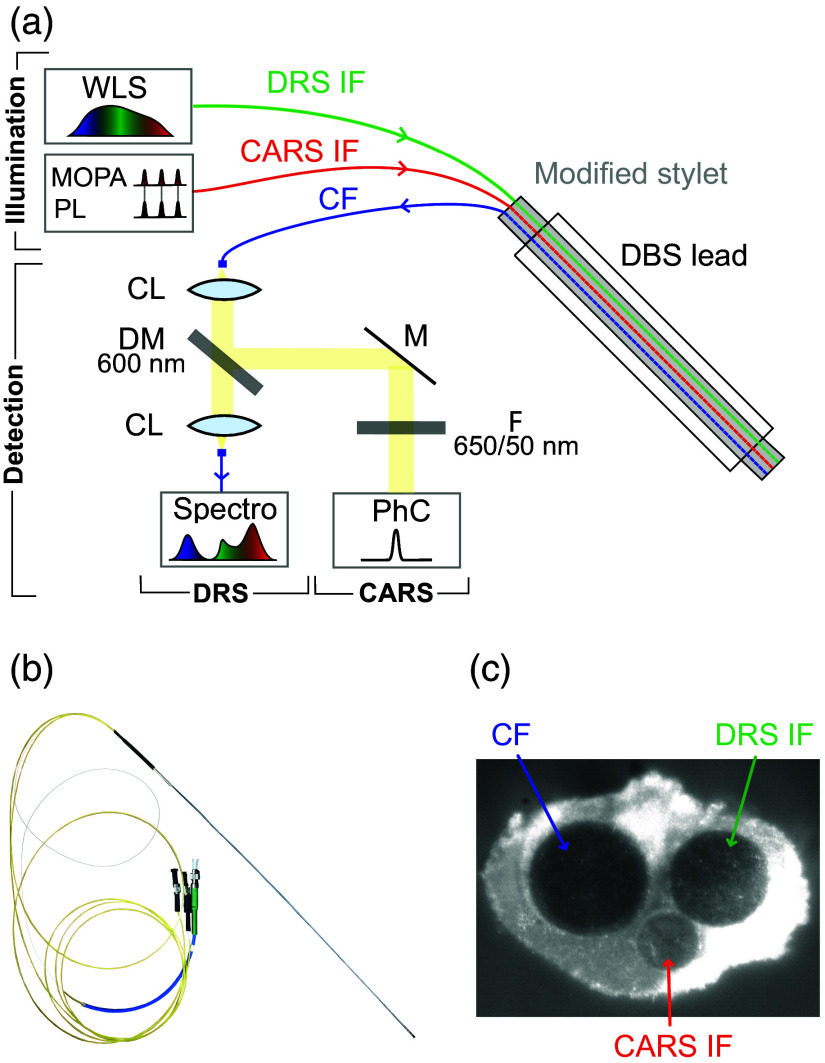
Optical probe and setup for illumination and collection for both CARS and DRS. (a) Schematic diagrams of the probe and the acquisition setup. (b) Photograph of the optical probe composed of a Medtronic 3389 DBS lead containing a modified stylet (metallic tube) and three optical fibers enclosed. (c) Photograph of the tip of the probe with the illumination and collection fibers visible. IF, illumination fiber; CF, collection fiber; CL, collimation lens; M, mirror; F, filter; WLS, white light source; MOPA, master oscillator amplifier; PL, programmable laser; Spectro, spectrometer; PhC, photon counter. For the left hemisphere acquisitions (without CARS), the CF was connected directly to the spectrometer to bypass the DM.

A custom-built probe composed of two optical fibers for signal emission and one for signal collection was built by inserting the fibers into a stainless steel tube instead of the standard tungsten stylet [Fig. 2(c)]. This modified hollow-core stylet has similar rigidity to the original stylet. The probe was then inserted into a DBS lead (model 3389, Medtronic, Dublin, Ireland) with a cut-off tip to provide an exit point for the fibers that emit light downward and away from the probe tip. The lead was then fixed to the micromanipulator, allowing optical measurements to be acquired along the insertion tract. More details on the DRS and CARS system can be found in the Supplementary Material.

A burr hole was drilled into the skull to access the brain. All spectral acquisitions began 40 mm before the target and continued for another 10 mm past the target, for a total length of 50 mm for each trajectory. CARS measurements were collected in the right hemisphere for a total of three trajectories (R-STN, R-GPi, and R-OFF), and DRS measurements were obtained using the same targets but in the left hemisphere (L-STN, L-GPi, and L-OFF). Since CARS measurements had a long acquisition time (8 s), the insertion step size was set to 1 mm to minimize the overall duration of the surgery. For the DRS measurements, the acquisition time was short (500 ms), and therefore, the insertion step size was reduced to 500  μm.

After completing the measurements, the probe was removed, and a narrow glass capillary was inserted into the hole created by the probe using the exact same angles. This was done so the trajectories would be visible on the postoperative MRI.

### 2.3 Brain Extraction and Tissue Manipulation

**Fig. 3:**
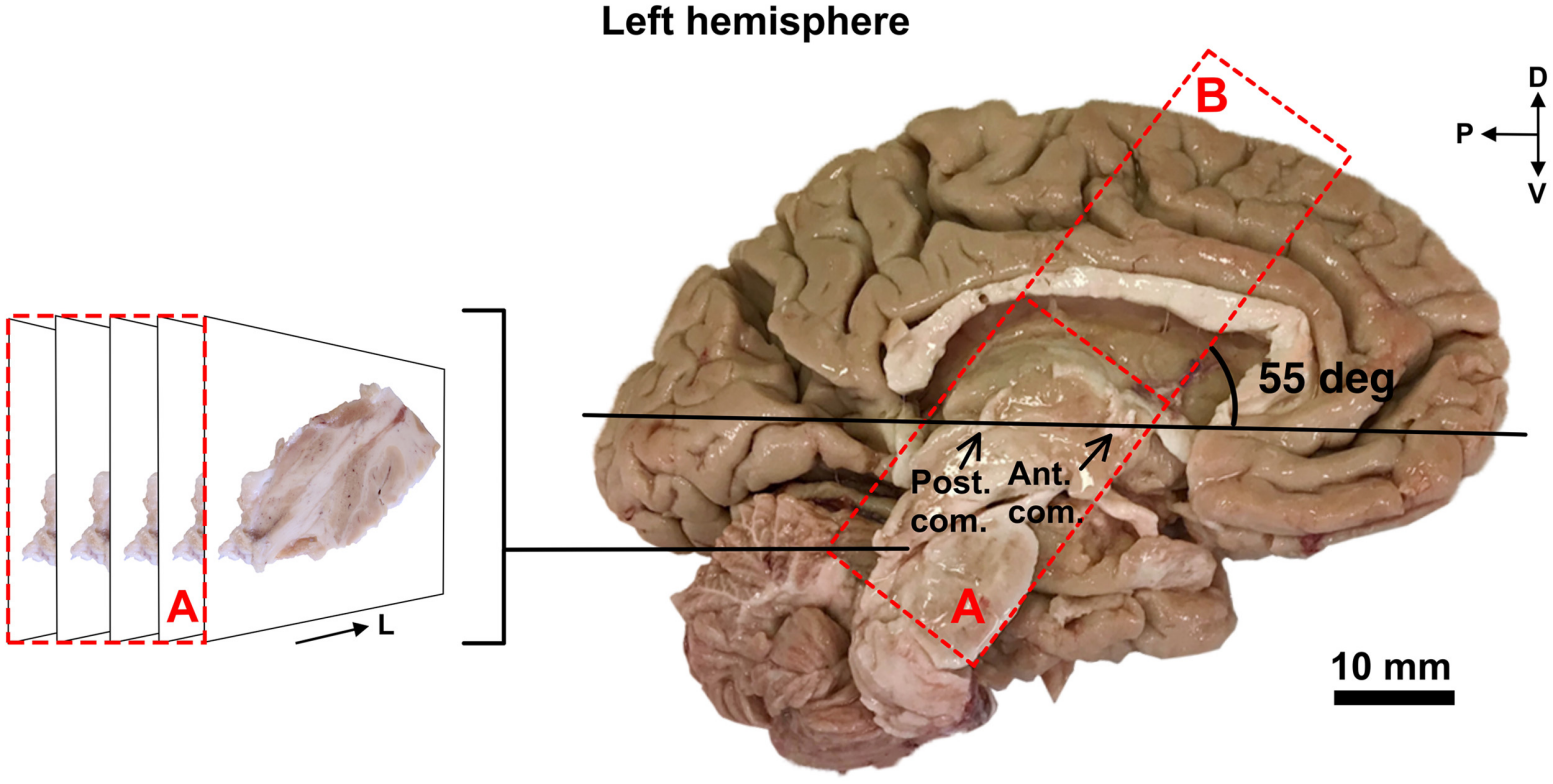
Dissection of the left hemisphere of the brain. In each hemisphere, the region of interest was cut into two blocks of tissue (A and B, defined by red dashed lines). The cuts were made at a 55 deg ring angle from the anterior-posterior commissure plane (shown in black) corresponding approximately to the ring angle used for the six trajectories. Each block was then fixed for two more days in paraformaldehyde and cut into 50  μm-thick sections using a freezing microtome. While cutting, a picture of each histological section was taken. Only the images from block A were used for analysis. D, dorsal; V, ventral; P, posterior; L, lateral.

After the surgery, 13 days postmortem, the brain was extracted and fixed by immersion in 4% paraformaldehyde at 4°C for 2 days. The brain was then cut along the midline into the two hemispheres. For each hemisphere, coronal cuts were made along a 55 deg ring angle taken from the anterior-posterior commissure plane to provide a block of tissue containing the six trajectories. A third cut was made to separate the basal ganglia from the cerebral cortex. A schematic of the cuts and sections obtained is shown in Fig. 3. These blocks of tissue were fixed for 2 more days in 4% paraformaldehyde and then stored at 4°C in 0.1 M PBS containing 15% sucrose and 0.1% sodium azide. The blocks were sliced with a freezing microtome into 50  μm-thick transverse sections, which were serially collected and preserved at −20°C in an antifreeze solution composed of 30% glycerol and 30% ethylene glycol diluted in PBS. A top-view image of the block was recorded every time a transverse section was collected. Since some insertion trajectories were not exactly parallel to the cutting plane, stitching several images was sometimes necessary to visualize the complete insertion tract.

### 2.4 Tissue Identification along the Insertion Tract via Barcodes

Identification of the tissue type (WM, GM) at each point along the insertion tract produces what we call a barcode, which provides the specific spatial sequence of tissue along the path of the probe as approximated by the modality. One barcode was produced for each modality used in this study: histology and optical spectroscopy. The histological barcodes (HISTO) were used as references for the optical barcodes. Sections 2.5 and 2.6 explain how we extracted the barcodes for each modality and trajectory.

### 2.5 Visual Tissue Identification on Histological Brain Slices

From the block of tissue containing the basal ganglia and the thalamus (block A in Fig. 3), sections in which lead tracts were visible were selected for further analysis. Different brain nuclei and brain regions were easily identified from the previously taken images using a brain atlas. As some brain regions are predominantly composed of WM or GM, identifying brain regions helped us determine the tissue type along the insertion tracts. When no specific brain structure could be clearly identified, the tissue type was classified based on the color of the tissue. Each section of the trajectory was classified as being either WM or GM. The results were then compiled to generate a unique barcode for each trajectory. This barcode serves as a ground truth for comparing the results of the spectral analysis.

### 2.6 Tissue Identification Using Spectral Analysis

A spectrum is a high-dimensional dataset where each level of intensity is considered an independent measurement. In trivial experiments (e.g., spectroscopy of clear liquids), a simple peak identification strategy is sufficient to unambiguously recognize a material. Here, that is not the case: there is no clear, obvious signature that can be easily observed. Therefore, CARS and DRS spectral data were analyzed using a dimensionality reduction technique called PCA and the k-means clustering technique to determine whether the probe was passing through WM or GM as it was inserted into the brain. PCA is a mathematical technique that reduces the complexity of a high-dimensional dataset using the eigenvectors of the covariance matrix as a new basis set. It identifies the most important patterns by finding a new set of variables, called principal components (PCs), that capture and quantify most of the variation in the original dataset. We applied PCA (with centering but without normalization) on the spectra for each tract. Then, the reduced dataset was used as an input in the k-means clustering technique to separate the data into k groups, of which there were two in our case. We interpreted the two groups as being WM and GM. From these results, six unique barcodes composed of either WM or GM were generated and compared with those obtained from the brain slices. More details about the spectral analysis methods are presented in the Supplementary Material.

## 3 Results

**Fig. 4:**
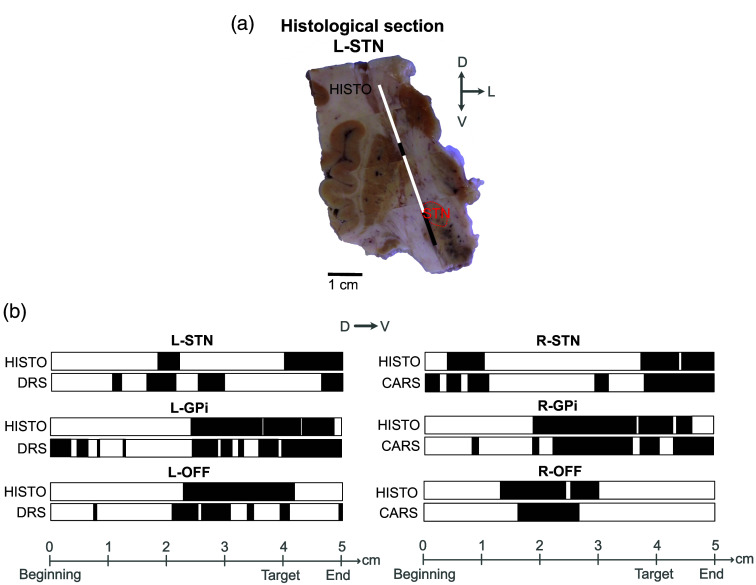
WM/GM discrimination in tissue is based on histology and optical spectra analysis using PCA and k-means clustering. (a) Example of barcode generation on a histological section where the trajectory for L-STN is visible. D, dorsal; L, lateral; V, ventral. (b) Barcodes from histology (HISTO) and spectral acquisitions (CARS or DRS) along the six insertion trajectories showing WM (white bars) and GM (black bars).

We were able to acquire optical spectra along six trajectories in a postmortem human brain. The tissues were identified as WM or GM to produce modality-specific barcodes, as presented in Fig. 4. The histological cut showing the L-STN tract is presented in Fig. 4(a) The comparison of all barcodes for each trajectory is presented in Fig. 4(b). Tissue identification was performed for all the optical spectra along one trajectory using PCA and k-means clustering (see the Supplementary Material). We noted that the first principal component (PC1) for individual trajectories explained over 98% of the data variance between the spectra. This means that PC1 alone could be sufficient for constructing the optical barcode. The barcode correspondence between histology and DRS is 73% on average, whereas it reaches 82% for histology and CARS.

## 4 Discussion

This work demonstrates the first successful attempt at acquiring CARS and DRS spectra of brain tissue for human DBS surgery. The DRS and CARS spectra from the optical probe can adequately differentiate WM and GM, as presented in Fig. 4(b). The optical measurements generally agree with the tissue classification from the HISTO barcodes for the same trajectory. However, small differences between the HISTO and optical barcodes remain. Due to the long postmortem delay and the lack of tissue fixation, the insertion of the optical probe may have caused brain compression and shifts. This may in turn lead to incorrect associations between tissue identification and the probe position, as well as mismatched reference and optical barcodes. In addition, some basal ganglia components, such as the striatum, the GPi, and the GPe, contain bundles of myelinated axons (WM) that travel through the GM. Therefore, these brain regions could be interpreted as mixed matter. We voluntarily decided to use two clusters instead of three (to include mixed matter) or a continuum of gray intensity to produce the barcodes, as identifying the tissue type on the histological slices from tissue color would become very arbitrary. Since our analysis clustered the data into two groups and did not consider the mixed matter, both the histology and optical identification could have incorrectly labeled the mixed matter as WM or GM. Although the CARS and DRS barcodes for each trajectory have similarities, we did not expect them to be identical since the entry points on the surface of the brain are different, as presented in Fig. 1(b). Moreover, the integration volume for DRS is $∼1\;{\mathrm{mm}}^{3}$ and only a few cubic micrometers for CARS, directly at the fiber tip. CARS is more sensitive to local features that could influence the signal, whereas DRS integrates over a broader spatial range, which could impact the resolution. Despite these shortcomings, comparing all three barcodes (STN, GPi, and OFF) for one optical modality (either DRS or CARS) shows visible differences, suggesting that the tissue composition for a specific trajectory is unique and can be recognized by our optical probe.

Based on what has been shown in the literature, we expected that comparing the optical barcodes with MRI data would not yield conclusive results. Indeed, Kobayashi et al.^30^ showed that MRI signal intensity changes in postmortem brain tissue compared with live tissue, especially in the basal ganglia, the region of interest in our work. Nevertheless, we analyzed the preoperative MRI scan and produced barcodes based on the pixel intensity value along each trajectory (see the Supplementary Material). As expected, the contrast for basal ganglia structures is very low, and T1-weighted MRI alone does not allow for an accurate WM/GM tissue classification. Furthermore, the MRI sequences used in this work are those used in the normal DBS surgery protocol at Hôpital de l’Enfant-Jésus to maximize target contrast and were not optimized for the present work. Other MRI sequences may be more appropriate for differentiating WM and GM. Ultimately, this identification of brain regions relies on the surgeons’ interpretation of the whole image and their knowledge of neuroanatomy and not just on pixel intensity along the tract. Consequently, we used the HISTO barcodes instead of the MRI barcodes as a ground truth to compare with the optical barcodes, but we plan on investigating optimal MRI sequence parameters for future measurements.

Our experiment presents a proof-of-concept of optical measurements for tissue identification in the human brain. More measurements need to be collected in clinical trials to assess the variability of the barcodes for specific targets and build a tissue spectra dataset for eventual real-time classification. Our group is also investigating the use of other modalities, such as polarization-sensitive optical coherence tomography, as it measures the birefringence of the myelin.^31^ We hypothesize that with more data and advanced analysis algorithms, optical barcodes could reflect MRI data. Our tool could eventually provide real-time insights into lead insertion accuracy as the neurosurgeon performs the procedure.

**Fig. 5:**
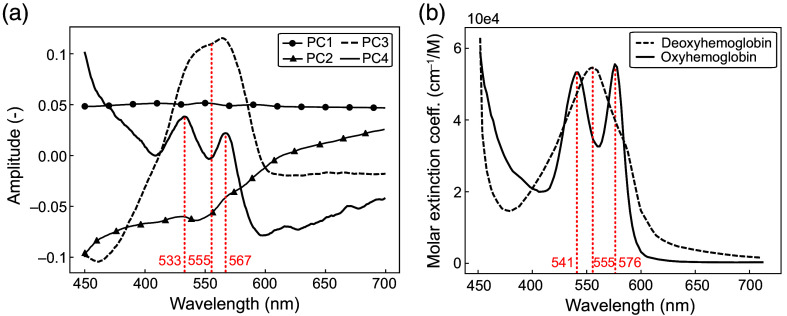
(a) Four first PCs obtained with PCA for the L-OFF trajectory. For the L-OFF trajectory, PC1 explained 98.937% of the variance of the spectral dataset, and PC2, PC3, and PC4 explained ∼0.09%, 0.01%, and 0.002%, respectively. (b) Deoxyhemoglobin and oxyhemoglobin absorption spectra obtained from Ref. 33.

The findings of this research indicate that DRS is a simple and minimally invasive optical method that could provide additional insight during DBS lead insertion. PCA revealed that tissue identification is mostly accomplished by PC1 alone. The first four PCs from the L-OFF trajectory (DRS) are shown in Fig. 5(a). PC1 for the L-OFF trajectory can be visualized as a straight line across all wavelengths, showing that the variance between the spectra acquired along the trajectory does not depend on the spectral information. This indicates that tissue identification is obtained only by the difference in light intensity that is scattered by the tissue. This observation is consistent with the inherent properties of brain tissue, where WM scatters more light than the darker GM tissue. It also suggests that measuring the intensity of the light reflected at a single wavelength in the visible range, from the tissue in front of the DBS lead, could be sufficient to differentiate WM and GM using the analytical method we provided here.

Considering the wealth of information that is often encoded in an optical spectrum, looking at other PCs beyond PC1 might have the potential to improve tissue identification. The four first PCs are presented in Fig. 5(a). The absorption spectrum of blood (deoxyhemoglobin and oxyhemoglobin) is shown in Fig. 5(b) for comparison. The two peaks observed in PC4 approximately correspond to the two peaks found in the oxyhemoglobin absorption spectrum. The wider peak in PC3 is approximately at the same wavelength as the main peak found in the deoxyhemoglobin absorption spectrum. However, the literature showed that oxyhemoglobin concentration decreases by 90% within 7 h of death.^32^ Therefore, it is not possible to conclude whether the optical probe detected very low concentrations of oxygenated blood or another unknown compound. Nevertheless, considering that oxyhemoglobin and deoxyhemoglobin are abundant in living subjects, it can be inferred that the spectroscopic measurements might reveal the presence of blood in the brain when inserting the optical probe. This could help prevent hemorrhage and further refine tissue identification. However, since this experiment was conducted on a postmortem brain, it was not possible to confirm the plausibility of this hypothesis. Finally, PC2 might display the wavelength dependence of scattering and absorption in biological tissue.

CARS showed promising results in tissue identification for PC1, which represents the intensity of the myelin signal in the tissue. However, attempts to use subsequent PCs for the analysis were unsuccessful despite using 10 mW of power and 8-s acquisitions at each depth along the trajectories. Indeed, only a very weak signal was detected, and the signal-to-noise ratio was poor, revealing essentially no visible features in the spectral decomposition. Similar to DRS, this means that our CARS tissue identification, which is adequate when compared with HISTO barcodes, was done simply with intensity and no spectral data were necessary. However, simple improvements could be made to our system to significantly enhance the CARS measurement quality. For example, since the laser wavelength is swept, the system could target specific vibrational modes that differentiate between WM and GM, such as 2845 or $2880\;{\mathrm{cm}}^{-1}$ for ${\mathrm{CH}}_{2}$ and $2916\;{\mathrm{cm}}^{-1}$ for ${\mathrm{CH}}_{3}$, which are abundant in myelin^27^. This way, a spectrum would only have a handful of relevant points, which would increase the signal per wavelength with the same acquisition time by more than one order of magnitude. With this simple change, our probe could acquire a spectrum in under a second, which could not only speed up the process but also provide more spectral information to improve our tissue identification. Other technical improvements, such as optimizing the probe design for more efficient signal collection by adding collection fibers, could increase the signal-to-noise ratio.

## 5 Conclusion

For the first time, we have acquired CARS and DRS spectra in the brain of a human cadaver and have demonstrated how they can be used to identify tissue. The spectra were acquired using a custom-built optical probe that was enclosed in a commercial DBS lead. The optical probe was then inserted in the brain, targeting three specific regions in the right and left hemispheres, for a total of six insertions. We observed that a barcode obtained from PCA and k-means clustering along a distinct trajectory appears unique. Using spectral data for brain tissue identification, we hope that the position of the lead could be followed in real time during its descent into the brain. In addition, DRS might be able to identify the presence of blood in the tissue to provide further guidance and prevent hemorrhages during lead insertion.

In the near future, more work is needed to refine the optical system to obtain higher-quality spectral acquisitions and to design a probe that is suitable for trials in living patients. A rigorous strategy to map the optical barcode onto the preoperative MRI scans with proper acquisition parameters will also be necessary for the success of the system. Nevertheless, we believe that our optical tool has the potential to significantly improve the outcomes of DBS surgeries by assessing the placement of the stimulation lead directly while it is inserted in the brain.

An expected barcode would be constructed from the preoperative MRI sequence according to the planned trajectory. During the stimulation lead insertion, a barcode from spectral acquisitions would be produced in real time and directly compared with the MRI barcode. By comparing the two barcodes, the system would inform the neurosurgeon about the placement of the DBS lead. When used alongside MERs, our optical probe would give the surgeon additional confirmation that the stimulating lead has reached the target as it was planned. Eventually, our tool could replace MERs and reduce total surgery time. In health centers where MER is not practiced and the patient is under general anesthesia, our optical probe would fill the need for a tool that confirms the lead placement by providing immediate feedback, thus improving patient outcomes.

## Supplementary Material



## Data Availability

All the data obtained from this experiment are publicly available at https://www.dccmlab.ca/lab-documentation/.
