# Supplementary material for: Human brain tissue identification using coherent anti-Stokes Raman scattering spectroscopy and diffuse reflectance spectroscopy for deep brain stimulation surgery
Source: Neurophotonics. 2024 Jun 12;11(2):025006. doi: 10.1117/1.NPh.11.2.025006 (PMC11167480; doi:10.1117/1.NPh.11.2.025006)
Supplement: Supplementary file 1 [file NPh_011_025006_SD001.pdf]

Supplementary Material

Human Brain Tissue Identification Using Coherent Anti-Stokes Raman Scattering Spectroscopy and Diffuse Reflectance Spectroscopy for Deep Brain Stimulation Surgery

Sébastien Jerczynski[a, b], Mireille Quémener[a, b], Valérie Pineau Noël[a, b], Antoine Rousseau[a, b], Elahe Parham[a, b], Alexandre Bédard[a, b], Shadi Masoumi[a, b], Thomas Charland[a, b], Anthony Drouin[a, b], Jonathan Roussel[a, b], Valérie Dionne[a, b], Thomas Shooner[a, b], Anaïs Parrot[c], Mohamad A Takech[d], Éric Philippe[d], Damon DePaoli[a, b], Léo Cantin[e], Martin Parent[a], Daniel C. Côté[a, b, *]

[a]CERVO Brain Research Center, 2301 avenue D'Estimauville, Québec, Canada, G1E 1T2

[b]Centre d'optique, photonique et laser, 2375 rue de la Terrasse, Québec, Canada, G1V 0A6

[c]Centre Hospitalier de l'Université Laval, CHU de Québec-Université Laval, 2705 boulevard Laurier, Québec, Canada, G1V 4G2

[d]Laboratoire d'anatomie, Faculté de médecine de l'Université Laval, 1050 avenue de la Médecine, Québec, Canada, G1V 0A6

[e]Hôpital de l'Enfant-Jésus, CHU de Québec-Université Laval, 1401 18e rue, Québec, Canada, G1J 1Z4

[*]dccote@cervo.ulaval.ca

1 Swept Coherent Anti-Stokes Raman Scattering

CARS measurements were acquired only in the right hemisphere with a 1-mm insertion step size, for a total of 3 trajectories. The laser system was a custom master oscillator power amplifier (MOPA) pump laser at 792 nm synchronized with a rapidly tunable programmable laser (PL) sweeping from 1020 to 1044 nm (Halifax Biomedical Inc). The wavelength ranges were chosen to interrogate specifically the high wavenumber region of the Raman spectrum (2800-3050 cm-1). Both lasers had a pulse width of 25 picoseconds and a 40 MHz repetition rate that was carefully aligned in space and time to optimize CARS signal generation from the tissue. The two lasers were sent through a single-mode, FC/AC terminated fiber (780HP, Thorlabs) in which the collimated light was sent through an array of signals. A custom-built probe composed of a single-

mode fiber for CARS laser delivery, a multimode fiber (FG105UCA, Thorlabs) for DRS light delivery, and a multimode fiber (FG105UCA, Thorlabs) for DRS and CARS signals collection was built and inserted inside the modified stylet. This stylet is located inside the DBS electrode used during the surgery. After signal collection, to separate DRS and CARS signal, a 600-nm dichroic mirror (TECHSPEC 600-nm Dichroic Highpass, Edmund Optics) was used. Finally, for the CARS signal, bandpass filters centered at 650 nm were used to limit the detection of the laser light. The CARS signal was recorded using a photon counter detector (H8259-02, Hamamatsu) combined with custom-built software programmed in MATLAB, which decoded the temporally-encoded CARS signal from the photon counter using the timing outputs from the laser system to plot the calculated spectra after the acquisition.

2 Diffuse reflectance spectroscopy

DRS measurements were collected on the left hemisphere. A fiber-based, white light source was used as the illumination component of the DRS through the delivery fiber of the custom-built probe. For left-hemisphere measurements, the collection fiber of the probe was directly plugged into a commercially available UV-VIS spectrometer (USB-4000, Ocean Optics) and the data were acquired using the software provided by the manufacturer.

3 Spectral analysis

The spectral analysis process is shown in Fig. S1. Because the CARS signal was very noisy, CARS data were first pre-processed using a Savitzky-Golay filter (Fig. S1, step 2). Then, PCA was performed on all data for each trajectory to reduce the dimensionality of the datasets using the sklearn Python package (Fig. S1, step 3). To identify the most relevant PCs to retain for subsequent analysis, the explained variance ratio of each PC was determined (Fig. S1, step 4). The PCs with the highest percentage of explained variance were conserved and used for clustering. The k-means clustering algorithm was applied to separate all the data into 2 groups that we interpreted as being either WM or GM (Fig. S1, step 5). K-means algorithm separates the data points in k groups by assigning them to the nearest centroïd through iterations. Note that due to the long

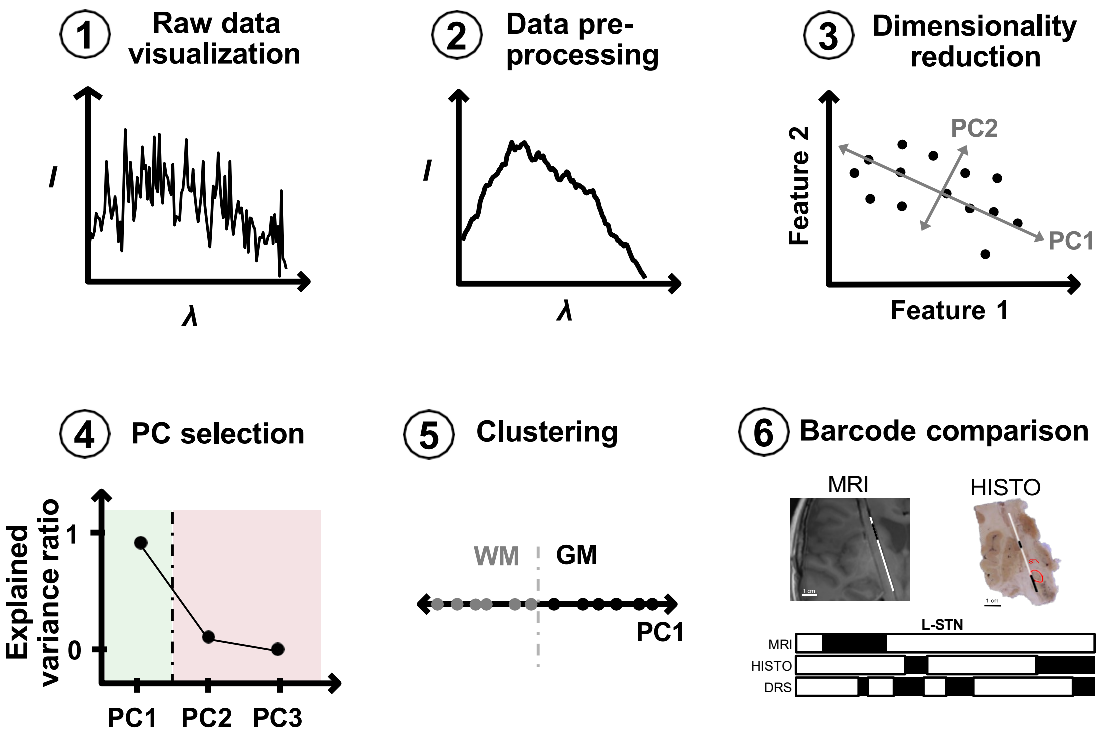

Fig S1 Spectral analysis pipeline followed in this study. Only CARS spectra were pre-processed using a Savitzky-Golay filter. A PCA algorithm using the sklearn Python module was applied on all spectra from each trajectory to reduce the dimensionality of the datasets. The first PCs explaining most of the variance of the dataset were retained for the rest of the analysis. Then, the k-means clustering method was performed to separate all data into 2 groups, which were interpreted as either WM or GM. The results were compared to the brain slices barcodes.

post-mortem delay, it was practically impossible to identify tissue types on histological sections with greater precision than white or gray matter. Adding a third category, mixed matter, would have been ideal. However, in our case, the identification would have been too subjective to make a fair comparison with spectral and MRI data. This is why we decided to use only two groups for the k-means algorithm. Finally, the results from this classification were compared to the ground truth from brain slice visualizations (Fig. S1, step 6). As an example, a visualization of the first 4 PCs for CARS and the PC1 score in the ROFF trajectory are presented in Fig. 4

4 Magnetic resonance imaging analysis

Numerous methods were explored and tested to obtain suitable barcodes from the MRI scans. We first produced an MRI barcode for each trajectory by analyzing the pixel intensity levels in the pre-

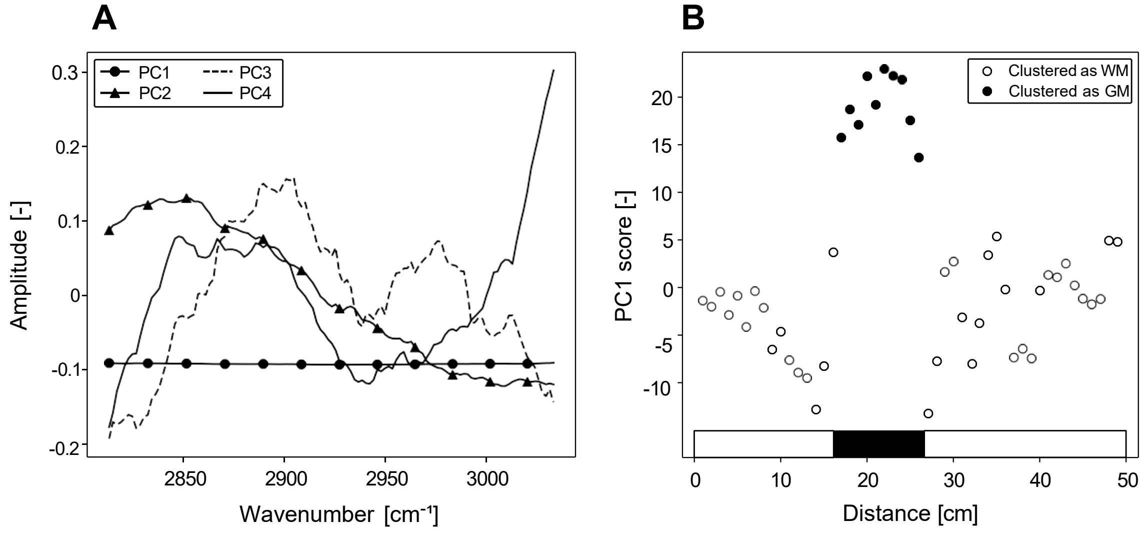

Fig S2 A: The first 4 PCs of the CARS spectra acquired in the ROFF trajectory. B: Score plot of PC1 in the ROFF trajectory. The optical barcode generated with the k-means clustering algorithm is also shown.

operative images. On the T1-weighted postoperative scan, the insertion coordinates were identified on each transverse plane by examining the visible capillaries in the images. The preoperative scan was then coregistered to the postoperative scan using the Advanced Normalization Tools (ANTs) software. Briefly, linear and nonlinear transformations were applied to guarantee proper coregistration since the brain shifted and changed shape slightly after the surgery.[1] Tissue analysis was performed on the preoperative image according to the coordinates obtained from the registration. By identifying the pixel intensity level of obvious GM regions (such as gyruses) and WM regions (such as the corpus callosum), we could estimate a WM-GM threshold by selecting the pixel intensity level in the middle. We focused on doing this analysis only with the T1-weighted modality, because we have complete scans of the brain, while T2-weighted and FLAIR were used to scan only sections of the brain. The resultant barcodes are presented in Fig. S3 as Patient MRI. We can see that the pixel intensity level from the preoperative T1-weighted scan is not a good indicator of the tissue type, as the targets of interest of this study (STN and GPi) are not visible due to a lack of contrast. As an alternative, we registered the widely known T1-weighted scan CIT168[2] to the postoperative T1-weighted MRI scan of our patient. In this case, we removed the skull of the postoperative T1-weighted MRI scan to produce the registration. We used the ANTs skull-stripping tool with the IXI brain template to perform this task. The registration of the CIT168 on the postoperative T1-weighted MRI scan and the extraction of the pixel intensity levels in each trajectory

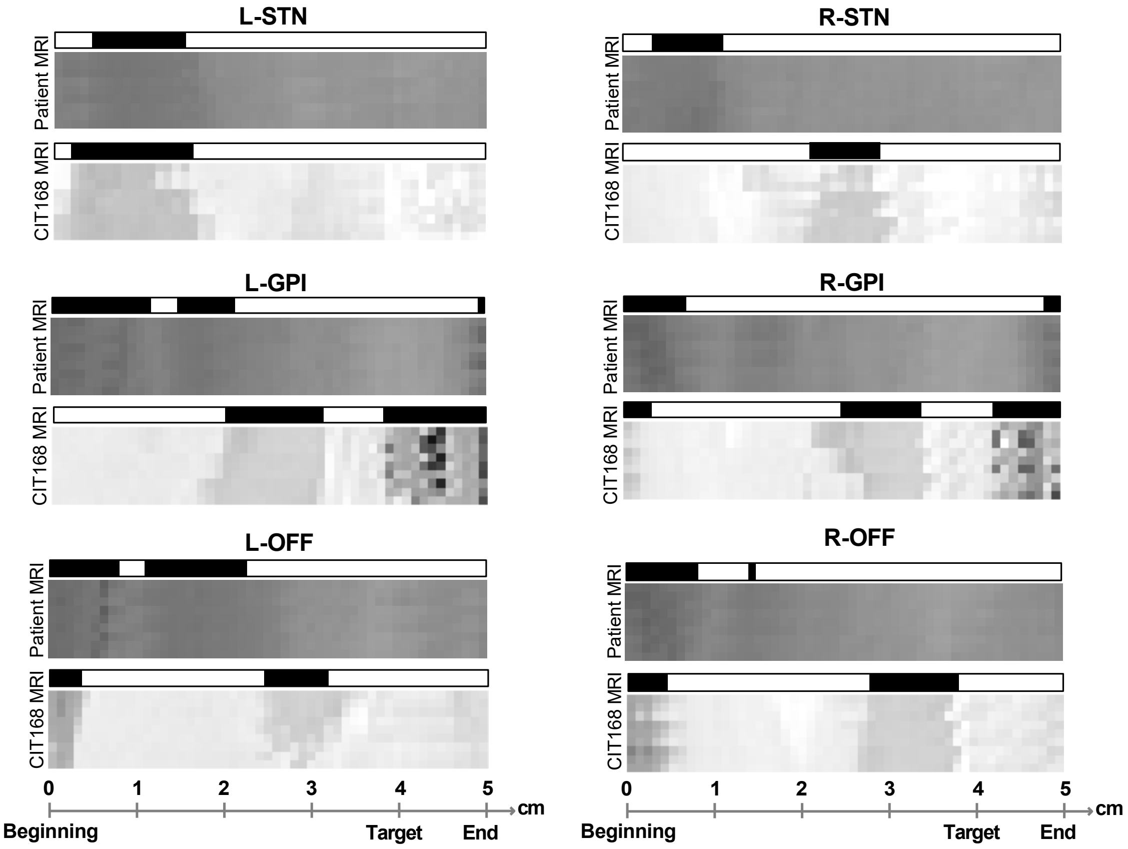

Fig S3 Visualization of the pixel intensity for each trajectory from the patient MRI and the CTI168 MRI atlas (both T1-weighted). At each depth, the intensity of the center pixel of the tract and 8 adjacent pixels are shown. The barcodes were generated using a threshold selected between the average intensities of different GM and WM structures.

were done as explained previously. The resultant barcodes are shown in Fig. S3 as CIT168 MRI. This method seems to produce barcodes with more contrast than the Patient MRI barcodes, but it is still not sufficient to discriminate WM and GM accurately. As an example, the STN (which is a gray structure) is not visible in both L-STN and R-STN tracts in Fig. S3, and appears as WM.

References

34. B. B. Avants, C. L. Epstein, M. Grossman, *et al.*, "Symmetric diffeomorphic image registration with cross-correlation: evaluating automated labeling of elderly and neurodegenerative brain," *Med. Image Anal.* **12**, 26–41 (2008).
35. T. JM and P. WM., "In vivo delineation of subdivisions of the human amygdaloid complex in a high-resolution group template," *Human Brain Mapping* **37** (2016).
